# P-760. Cutaneous Nocardiosis: Clinical Characteristics, Treatment Patterns, and Outcomes

**DOI:** 10.1093/ofid/ofaf695.971

**Published:** 2026-01-11

**Authors:** Maria Vega Brizneda, Cyndee Miranda, Eric Cober, Anisha Misra, Susan Harrington, Zachary Yetmar

**Affiliations:** Cleveland Clinic, Cleveland, OH; Cleveland Clinic, Cleveland, OH; Cleveland Clinic Foundation, Cleveland, OH; Cleveland Clinic Foundation, Cleveland, OH; Cleveland Clinic, Cleveland, OH; Cleveland Clinic, Cleveland, OH

## Abstract

**Background:**

Cutaneous nocardiosis usually occurs due to direct inoculation into the skin after trauma in immunocompetent individuals but can also be part of a disseminated disease in immunocompromised hosts. The comparative burden of isolated versus disseminated cutaneous involvement remains poorly defined. We aimed to characterize clinical presentation, management, and outcomes in patients with cutaneous nocardiosis.

**Table 1.**
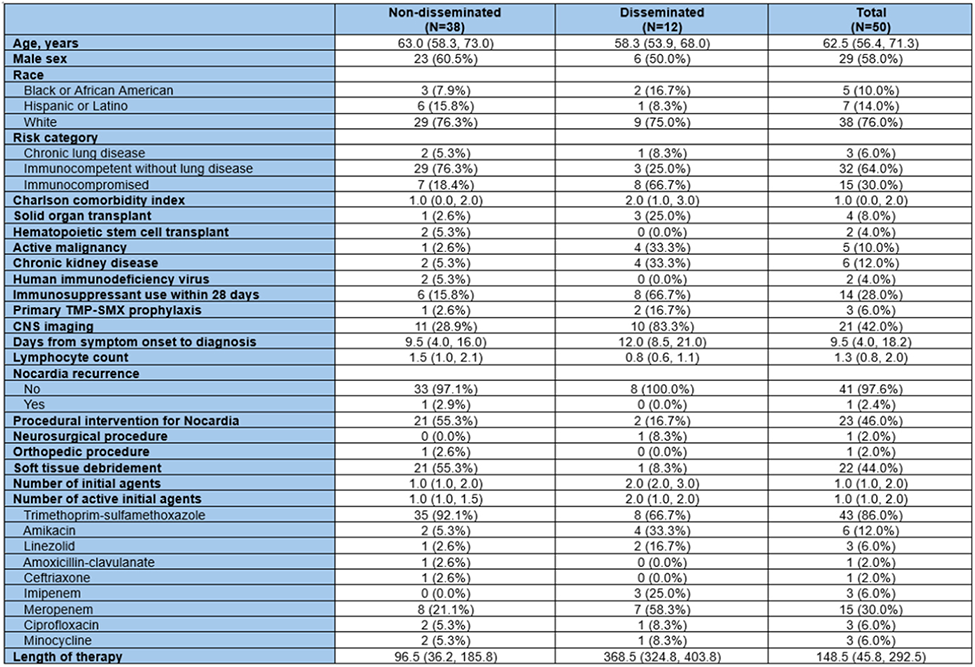
Characteristics of patients with cutaneous nocardiosis

Table 2.

**Figure:**
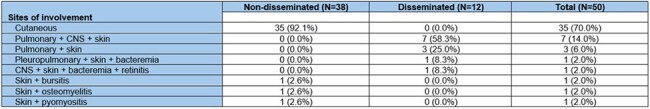

**Methods:**

We conducted a retrospective cohort study of adult patients diagnosed with cutaneous nocardiosis at a tertiary institution from January 1, 2010, to December 31, 2023. Patients were categorized as having either non-disseminated (n=38) or disseminated (n=12) disease. We compared baseline characteristics, immune status, treatment, and outcomes including one-year mortality and post-treatment recurrence.

**Results:**

Non-disseminated cases primarily occurred in immunocompetent individuals without lung disease (76.3%), whereas disseminated cases were predominantly among immunocompromised patients (66.7%, p=0.002). Solid organ transplant (25.0% vs 2.6%, p=0.038), active malignancy (33.3% vs 2.6%, p=0.009), and recent immunosuppressant use (66.7% vs 15.8%, p=0.002) were associated with higher rates of dissemination and these groups received significantly longer therapy (median 368.5 vs 96.5 days, p< 0.001) and a higher number of active agents. In the non-disseminated cases, procedural interventions, including soft tissue debridement, were more common (55.3% vs 8.3%, p=0.006). N. brasiliensis predominated in non-disseminated cases, while N. farcinica was significantly in dissemination (p< 0.001). Recurrence was rare (2.4%) with a median follow-up of 3.4 years post-treatment among survivors. One-year mortality occurred exclusively in the disseminated group (25.0% vs 0%, p=0.011).

**Conclusion:**

Cutaneous nocardiosis in immunocompetent patients typically presents as non-disseminated disease whereas disseminated cutaneous nocardiosis is strongly associated with immunosuppression and carries a worse prognosis, including increased mortality. Post-treatment recurrence is rare even with shorter courses of therapy. Recognition of divergent clinical characteristics may aid with diagnostic and therapeutic strategies.

**Disclosures:**

Susan Harrington, PhD, Bruker Daltonics, Inc: Grant/Research Support

